# A novel *de novo IL2RG* nonsense mutation in a pediatric patient with X-linked severe combined immunodeficiency

**DOI:** 10.3389/fgene.2025.1641956

**Published:** 2025-10-07

**Authors:** Lin Wang, Aiyan Ren, Yuanyuan He, Lu Wang, Sixi Wang, Xin Xie, Mei Tan, Yan Chen, Pei Huang, Zuochen Du

**Affiliations:** ^1^ Department of Pediatrics, Affiliated Hospital of Zunyi Medical University, Zunyi, China; ^2^ Guizhou Children’s Hospital, Zunyi, China; ^3^ Collaborative Innovation Center for Tissue Injury Repair and Regenerative Medicine of Zunyi Medical University, Zunyi, China

**Keywords:** IL2RG, X-SCID, lymphocyte, *Talaromyces marneffei*, T cell

## Abstract

**Objective:**

The study aims to describe the clinical manifestations, immunophenotype, and gene mutation characteristics of a child with X-linked severe combined immunodeficiency (X-SCID) caused by an *IL2RG* mutation (NM_000206.3; exon 2 c.216C > A, p.Cys72*), and to contextualize these findings through a review of reported cases of X-SCID associated with *Talaromyces marneffei* (*T. marneffei*) infection, highlighting the immunological and diagnostic relevance of the mutation.

**Methods:**

The clinical data of a child with X-SCID caused by an *IL2RG* mutation were retrospectively analyzed. Whole-exome sequencing and Sanger sequencing were used to identify the mutation, and flow cytometry was employed for immunophenotypic analysis to assess the impact of the mutation on immune function. A literature review was performed on reported cases of X-SCID associated with *T. marneffei* infection identified in PubMed and Scopus.

**Results:**

The patient was a 4-month-old male infant presenting with chronic diarrhea, recurrent fever, and anemia, with poor response to antibiotic treatment. Laboratory tests indicated *T. marneffei* infection, significantly reduced T lymphocytes and NK cells, and decreased levels of immunoglobulins (IgG, IgA, IgE). Genetic testing revealed a *de novo* hemizygous mutation in the *IL2RG* gene (NM_000206.3; exon2 c.216C > A, p.Cys72*), which has not been previously reported. Flow cytometric analysis showed a severe reduction in T lymphocytes, absence of CD8 ^+^ T cells, reduced NK cells, and a normal number of B lymphocytes. Six cases of *IL2RG* gene mutation combined with *T. marneffei* infection were identified in the literature, most showing fever, hepatosplenomegaly and respiratory tract infection.

**Conclusion:**

This case of X-SCID caused by a novel *de novo IL2RG* mutation expands the known mutation spectrum and highlights its immunological and diagnostic relevance. The review of reported cases of X-SCID with *T. marneffei* infection further clarifies genotype–phenotype correlations and supports improved recognition of such rare presentations.

## 1 Introduction

Inborn Errors of Immunity (IEI), historically referred to as Primary Immunodeficiency Diseases (PIDs), are a group of genetically heterogeneous disorders caused by defects in the immune system, leading to increased susceptibility to infections. Since 1970, the World Health Organization (WHO) has organized meetings and established the International Union of Immunological Societies (IUIS), which defined and classified “Primary Immunodeficiency Diseases.” Initially, 16 distinct immunodeficiencies were identified. Approximately every 2 years, the IUIS releases updated reports, refining the classification of IEI. In 2022, the IUIS updated its classification, maintaining the 10 major categories of IEI established in 2019 and incorporating 55 new monogenic defects along with one autoantibody phenotype, detailing their key clinical and laboratory features (Tangye et al., 2022). Given the involvement of hundreds of genes in IEI, clinical phenotypes are often not highly specific, making diagnosis based solely on phenotypes challenging. Consequently, most novel variants are identified through next-generation sequencing (NGS) technologies, with whole-exome sequencing (WES) and whole-genome sequencing (WGS) now considered the standard for identifying new pathogenic gene variants (Seleman et al., 2017; Meyts et al., 2016).

Severe Combined Immunodeficiency (SCID), one of the most devastating forms of IEI, is a genetically heterogeneous disorder characterized by arrested T lymphocyte development, accompanied by varying degrees of functional abnormalities in B cells and NK cells, leading to severe impairment of both cellular and humoral immunity (Al Sukaiti et al., 2021). Infants with SCID typically present with respiratory symptoms, chronic diarrhea, failure to thrive, and recurrent opportunistic infections within the first 2–6 months of life (Aluri et al., 2019). The incidence of SCID varies geographically. Epidemiological data indicate an estimated prevalence of 1.7 per 100,000 live births in the Greece-Turkey region (Michos et al., 2011). In Germany, a comprehensive analysis of SCID cases diagnosed in pediatric hospitals between 2014 and 2015, using stringent inclusion and exclusion criteria, estimated an annual incidence of 1.6 per 100,000 live births (Shai et al., 2020). In Israel, neonatal screening for SCID revealed a higher incidence of 1 in 29,000 live births (Lev et al., 2022). These findings highlight the importance of region-specific epidemiological studies and early diagnostic interventions for SCID.

X-linked severe combined immunodeficiency (X-SCID), caused by mutations in the interleukin-2 receptor gamma chain (*IL2RG*) gene, is one of the most common forms of SCID, accounting for approximately 50%–60% of cases (Mahdavi et al., 2021). Infants with X-SCID typically present with clinical symptoms within the first 2–5 months of life, characterized by recurrent opportunistic infections and reduced numbers and impaired function of T cells and/or B cells. Without treatment, most X-SCID patients succumb to infections within the first year of life (Jiang et al., 2024). Current therapeutic strategies for X-SCID include infection prophylaxis, immune replacement therapy, hematopoietic stem cell transplantation (HSCT), and certain gene therapies (Aranda et al., 2024). Although molecular genetic methods such as quantitative detection of T-cell receptor excision circles (TRECs) (Currier and Puck, 2021) and kappa-deleting recombination excision circles (KRECs) (Nakagawa et al., 2011) are available for SCID screening, clinical implementation of SCID screening and genetic testing remains limited (Boyarchuk et al., 2022). Additionally, due to the early onset and nonspecific clinical manifestations of X-SCID, clinicians often face challenges in accurately diagnosing immunodeficiency disorders at an early stage.

In this study, we describe the clinical manifestations, immunophenotypic characteristics, and diagnostic and therapeutic management of a patient with X-SCID harboring a novel *de novo IL2RG* mutation. We also provide a comprehensive review of all reported X-SCID cases combined with *Talaromyces marneffei* (*T. marneffei*) infection in the literature. The aim of this study is to describe the clinical and immunophenotypic features of a patient with X-SCID harboring a novel *de novo IL2RG* mutation, review all reported cases of X-SCID associated with *T. marneffei* infection, and provide insights to support early diagnosis, treatment, and screening in at-risk families.

## 2 Materials and methods

### 2.1 Patient

The subject of this study was a 4-month-old male infant. Clinical data were collected after obtaining informed consent from his legal guardian. This study was conducted in accordance with the principles of the Declaration of Helsinki and was approved by the Ethics Committee of the Affiliated Hospital of Zunyi Medical University.

### 2.2 Genetic sequencing analysis

Peripheral blood samples were collected from the patient and both parents for DNA extraction. Whole-exome sequencing was performed using NGS technology and Sanger sequencing (MyGenostics, Beijing, China) was applied for variant validation. Databases such as HGMD, ClinVar, PubMed, and Scopus were searched, and relevant literature was reviewed. Variants were classified according to the guidelines of the American College of Medical Genetics and Genomics (ACMG).

### 2.3 Flow cytometry

Peripheral blood mononuclear cells (PBMCs) of the patient were isolated and counted. The following fluorescently labeled antibodies were used for surface staining of T and B cells: anti-TCRαβ-PE (306708; BioLegend), anti-TCRγδ-Brilliant Violet 421 (331218; BioLegend), anti-CD3-PerCP (300326; BioLegend), anti-CD4-FITC (300506; BioLegend), anti-CD8a-Brilliant Violet 510 (301048; BioLegend), anti-CD27-APC (356410; BioLegend), anti-CD45RA-PE-Cy7 (304126; BioLegend), anti-CD19-APC (302212; BioLegend), anti-CD27-PB (302822; BioLegend), anti-IgD-AF488 (348216; BioLegend), anti-CD38-PerCP (303520; BioLegend), and anti-CD24-PE (311106; BioLegend). Stained cells were analyzed using a FACS Canto Plus flow cytometry (BD, United States) and data were processed using FlowJo software.

## 3 Results

### 3.1 Case presentation

A 4-month-old male infant with no family history of immunodeficiency was born via full-term cesarean section to healthy, non-consanguineous parents (G1P1). Birth history indicated nuchal cord and meconium-stained amniotic fluid, with a history of neonatal transfusion. Postnatal feeding involved a combination of breast milk and formula. At 3 months of age, the infant developed recurrent diarrhea (7–8 episodes daily), characterized by yellow-green loose stools with mucus. Symptomatic treatment reduced stool frequency but did not improve stool consistency. By 4 months of age, he exhibited recurrent fever and persistent diarrhea. Laboratory tests revealed anemia with a hemoglobin level of 85 g/L (reference range, 100–140 g/L). During hospitalization, anti-infective and supportive therapies were ineffective. After referral, due to the complexity of the condition, the family ultimately opted to discontinue treatment. Key clinical features throughout the disease course included recurrent fever, diarrhea, cough, progressive anemia, hepatosplenomegaly and generalized edema (Table 1).

**TABLE 1:** Summary of clinical manifestations of the patient.

Various systems	Clinical manifestations	Laboratory analysis	Treatment
Respiratory system	Bronchopneumonia	*T. marneffei* and *M. pneumoniae*	Antibiotic
Digestive system	Chronic diarrhea	Gram-negative bacteria++++	-
	Lactose intolerance	Urine galactose detection weakly positive	-
Blood system	Medium anemia	HGB 79 (median) g/L↓	Blood transfusion
Circulating system	Small atrial septal defect	Small atrial septal defect or patent foramen ovale A small amount of pericardial effusion	-
Immune system	Low IgG IgA	IgG 6.40 g/L↓	-
		IgA 0.02 g/L↓	-
Urinary system	Hypoproteinemia	Albumin 24.8 (median) g/L↓	Albumin infusion

HGB, hemoglobin.

### 3.2 Laboratory examination and inspection

**FIGURE 1:**
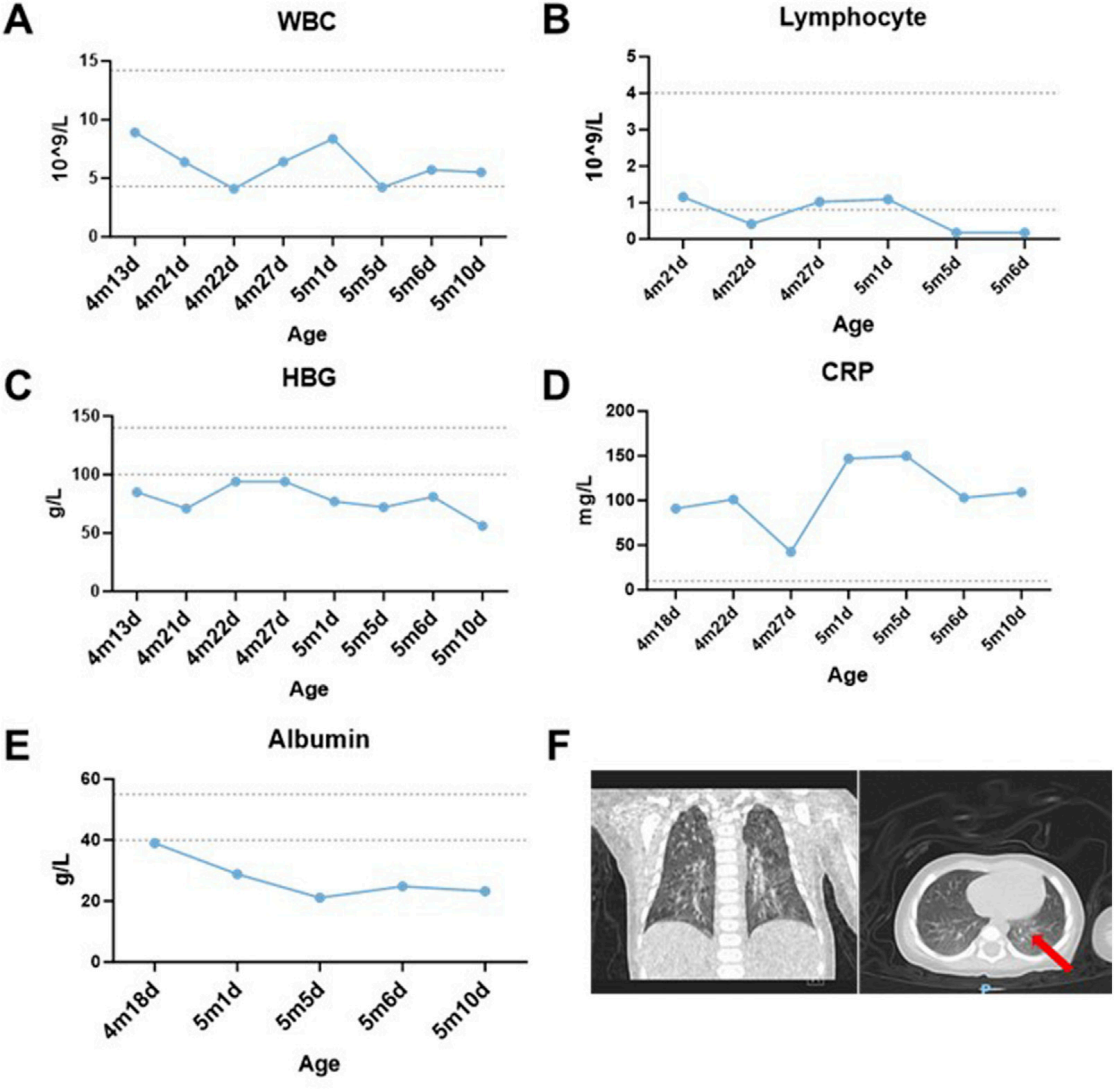
Laboratory examinations and results. **(A,B)** Dynamic changes of white blood cell (WBC) and lymphocyte counts in the patient over time. **(C)** Post-transfusion hemoglobin (HGB) levels failed to normalize despite red blood cell transfusions on 4m21d, 4m22d, and 5m5d. **(D)** Hypersensitive C-reactive protein (hs-CRP) fluctuated repeatedly during hospitalization, remaining around 100 mg/L except on 4m27d. **(E)** Temporal changes in serum albumin levels. **(F)** Coronal chest CT demonstrating scattered inflammatory infiltrates in bilateral lungs (red arrow). The ‘m’ indicates months, while the ‘d’ signifies day.

Continuous monitoring of routine blood parameters after admission revealed the following: although the white blood cell count remained within normal range, lymphocyte counts were markedly reduced (0.17–1.15 × 10^9^/L, reference 0.8–4.0 × 10^9^/L) (Figures 1A,B). Hemoglobin levels remained persistently below normal despite multiple erythrocyte transfusions (56–94 g/L, reference 100–140 g/L) (Figure 1C). A markedly elevated high-sensitivity C-reactive protein (hs-CRP) level suggested an ongoing infectious process. The observed decline in hs-CRP at 4 months and 27 days of age was attributed to a 3-day course of glucocorticoid therapy. Notably, the hs-CRP subsequently increased following discontinuation of corticosteroid treatment (Figure 1D).

The patient developed generalized edema accompanied by progressive hypoalbuminemia during hospitalization (21.1–39 g/L, reference 40–55 g/L) (Figure 1E). Imaging studies revealed bilateral bronchopneumonia on Chest CT (Figure 1F) and a small atrial septal defect or patent foramen ovale with left-to-right atrial shunt and minimal pericardial effusion on cardiac ultrasound. Immunoglobulin assays showed significantly decreased IgA (0.02 g/L, reference 0.72–4.29 g/L) and IgE (<5 IU/mL, reference <165 IU/mL), with IgG also below the normal range. Inflammatory factors, such as IL-6 (111.57 pg/mL, reference 1.18–5.30 pg/mL), were significantly elevated (Table 2). Urinalysis demonstrated weakly positive galactose. The patient presented with co-infection of *Mycoplasma pneumoniae* and *T. marneffei*.

**TABLE 2:** Immune function test results.

Items	Result	Units	References range
Immunoglobulins			
lgG	6.40	g/L	7.51–15.6
lgA	0.02	g/L	0.72–4.29
lgM	0.92	g/L	0.46–3.04
lgE	<5.00	IU/mL	<165.00
C3	1.20	g/L	0.79–1.52
C4	0.26	g/L	0.16–0.38
TH1/TH2 subgroup detection			
IL-2	0.43	pg/mL	0.08–5.71
IL-4	1.43	pg/mL	0.10–2.80
IL-6	111.57	pg/mL	1.18–5.30
IL-10	68.19	pg/mL	0.19–4.91
TNF-α	2.36	pg/mL	0.10–2.31
IFN-γ	4.84	pg/mL	0.16–7.42

The red indicates an increase, while the blue signifies a decrease.

During comprehensive anti-infective therapy, sequential administration of third-generation cephalosporins, carbapenem antibiotics (imipenem), and antifungal agents (voriconazole and amphotericin B lipid complex) was initiated. Supportive treatments included packed red blood cell transfusions for severe anemia, human albumin supplementation to correct hypoalbuminemia-associated edema, and intravenous immunoglobulin for immunomodulation. Post-transfer, the antimicrobial regimen was augmented with linezolid and levofloxacin, alongside invasive mechanical ventilation and continuous blood purification therapy. Despite multidisciplinary intensive care, the child exhibited persistent multi-organ dysfunction with no resolution of inflammatory markers or radiographic abnormalities. Due to progression to end-stage disease, the family elected to withdraw life-sustaining therapies.

### 3.3 Genetic testing and analysis

**FIGURE 2:**
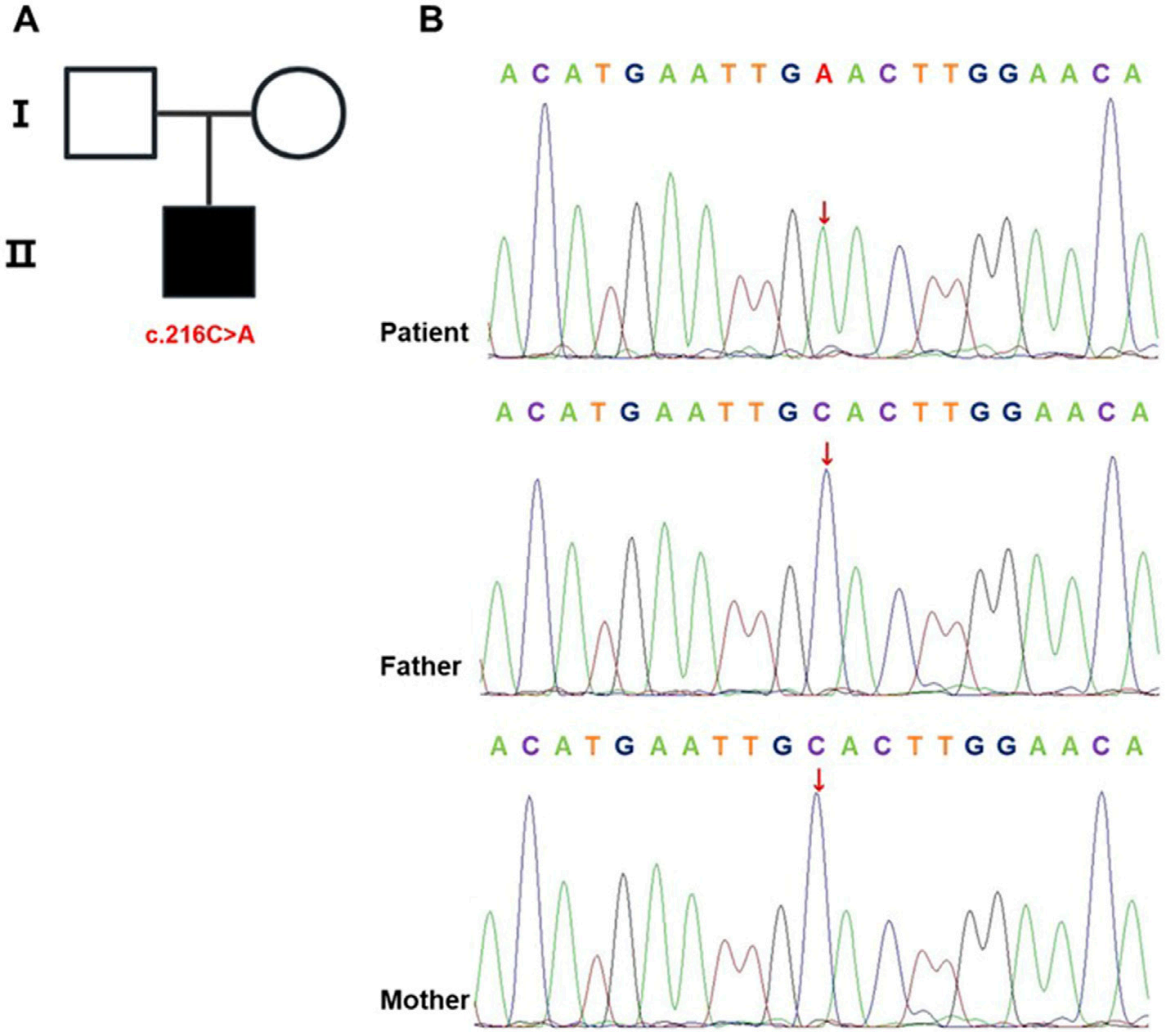
*IL2RG* mutation identified in the proband. **(A)** Pedigree chart. Males are represented by squares and females by circles, with the black-shaded square denoting the affected male carrying the mutation. **(B)** Sanger sequencing of *IL2RG* in the proband and his parents. The analysis identified a c.216C > A mutation in the proband, while this variant was absent in both parental samples.

Based on the patient’s postnatal history of recurrent bacterial infections, chronic diarrhea, and persistent hypoalbuminemia, an IEI was strongly suspected. Given the absence of similar clinical manifestations in either parent, the mutation was hypothesized to be *de novo*. WES of peripheral blood samples from the patient and both parents identified a nonsense variant in *IL2RG* (NM_000206.3, exon 2, c.216C > A, p.Cys72*). According to the ACMG/AMP variant interpretation guidelines, this variant met criteria for classification as pathogenic (PVS1 [predicted loss-of-function variant] + PS2 [*de novo* origin] + PM2 [absent in population databases]). Family pedigree analysis confirmed no familial recurrence (Figure 2A). The ClinVar database contained no prior reports associating this variant with disease. Sanger sequencing further confirmed its *de novo* origin (Figure 2B).

### 3.4 Immune function evaluation

Previous studies have shown that X-SCID caused by *IL2RG* mutations results in defective T lymphocyte development. We performed flow cytometry to quantify lymphocyte subsets in the patient’s PBMCs (Table 3). Results showed a markedly reduced total T cell count, with undetectable CD8^+^ T cells and an extremely low proportion of CD4^+^ T cells; residual T cells were predominantly naive and central memory. B lymphocytes showed an increased total count but were composed mainly of immature B cells, accounting for 96.10% of the population. The nonsense mutation in *IL2RG* caused severe T cell depletion, impairing T-B cell collaboration and leading to B cell differentiation arrest. Persistent bacterial and viral infections likely further contributed to B cell dysfunction.

**TABLE 3:** Lymphocyte subset profile of the patient.

Lymphocyte subsets	Percentage (%)	References range	Number (cells/μL)	References range
T cell	0.31 (L%)	54.28–71.67	3.38	2,179–4,424
CD8^+^T cells	0.00 (L%)	14.08–24.70	0.00	556–1,687
CD8^+^ Naïve	0.00 (CD8%)	68.90–94.60	0.00	503–1,276
CD8^+^ TEMRA	0.00 (CD8%)	0.02–9.61	0.00	0–133
CD8^+^ CM	0.00 (CD8%)	5.14–25.55	0.00	41–305
CD8^+^ EM	0.00 (CD8%)	0.10–4.95	0.00	1–70
CD4^+^T cells	0.03 (L%)	33.72–52.43	0.36	1,461–3,018
CD4^+^ Naive	25.00 (CD4%)	69.15–88.10	0.09	1,170–2,595
CD4^+^ TEMRA	0.00 (CD4%)	0.00–1.64	0.00	0–40
CD4^+^ CM	75.00 (CD4%)	10.11–28.20	0.27	213–647
CD4^+^ EM	0.00 (CD4%)	0.28–2.10	0.00	5–48
TCRαβ^+^DNT	0.00 (T%)	0.33–1.12	0.00	11–45
γδ T cells	5.26 (T%)	3.32–7.40	0.17	92–279
B cells	98.50 (L%)	17.34–36.03	1,073.65	734–2,265
Unswitched memory B	0.057 (B%)	0.99–4.71	0.61	12–54
Naïve B	96.10 (B%)	87.55–94.85	1,032.23	691–2,132
Transitional B	6.17 (B%)	15.05–29.95	66.27	136–464
Plasmablasts B	0.06 (B%)	0.60–3.95	0.61	6–42
NK cells	7.00	5.89–14.85	22.00	290–780
DPT cell	-	0.33–0.92	-	16–56
CD4^+^: CD8^+^	0.00	1.47–3.23	-	-

NA: Not Applicable. The red indicates an increase, while the blue signifies a decrease. L%” means “percentage of lymphocytes”. CD8% means “percentage of CD8^+^ T cells”. CD4% means “percentage of CD4^+^ T cells”. T% means “percentage of CD3^+^ T cells”. B% means “percentage of CD19^+^ B cells”. Naïve: cytotoxic T lymphocyte with differentiation markers: CD3^+^ CD8^+^ CD45RA^+^ CD27^+^ TEMRA: terminally differentiated effector memory: CD3^+^ CD8^+^ CD45RA^+^ CD27^−^ CD8^+^ CM: central memory: CD3^+^ CD8^+^ CD45RA^−^ CD27^+^ CD8^+^ EM: effector memory: CD3^+^ CD8^+^ CD45RA^−^ CD27^−^ Naïve: helper T lymphocyte markers: CD3^+^ CD4^+^ CD45RA^+^ CD27^+^ TEMRA: terminal effector memory differentiation: CD3^+^ CD4^+^ CD45RA^+^ CD27^−^ CD4^+^ CM: central memory: CD3^+^ CD4^+^ CD45RA^−^ CD27^+^ CD4^+^ EM: effector memory: CD3^+^ CD4^+^ CD45RA^−^ CD27^−^ TCR^−−^^+^ DNT: double negative T lymphocytes: CD3^+^ TCR^−−^^+^ CD4^−^ CD8^−^ Unswitched memory B cells: CD19^+^ CD27^+^ IgD^+^ Naive B cells: CD19^+^ CD27^−^ IgD^+^ Transitional B cells: CD19^+^ CD24^+^^+^ CD38^+^^+^ Plasmablasts: CD19^+^ CD24^−^ CD38^+^^+^

### 3.5 Summary of X-SCID cases combined with *T. marneffei* infection in the literature

**FIGURE 3:**
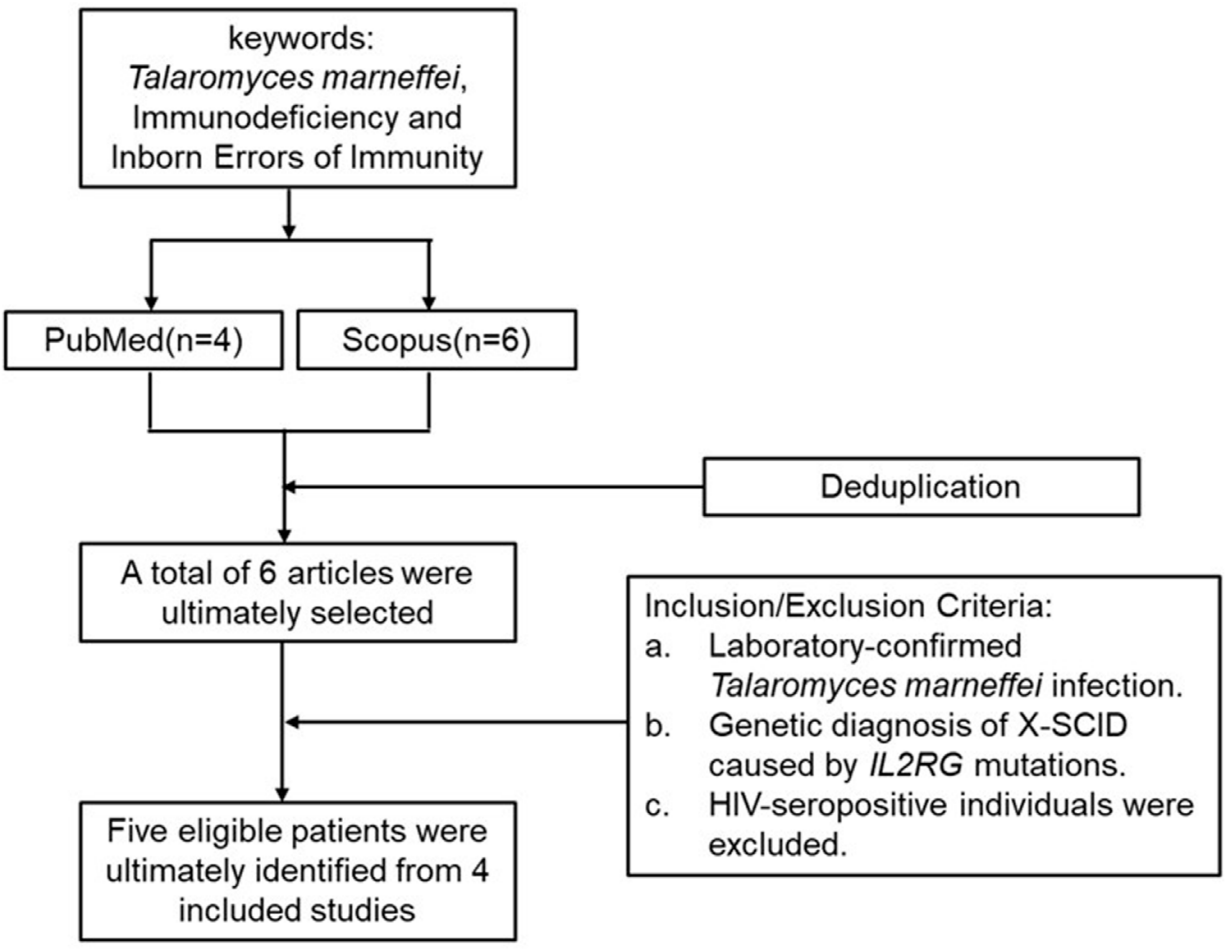
Literature search and selection process for reported cases of *IL2RG* mutation with *T. marneffei* infection.

In this case, *T. marneffei* was isolated from both bone marrow and peripheral blood cultures, with serological testing confirming of HIV seronegativity. An initial keyword search in PubMed and Scopus retrieved six articles. After applying predefined inclusion and exclusion criteria, five eligible patients were ultimately identified from 4 included studies (Figure 3). Clinical data from these five patients, together with the present case, were extracted and systematically analyzed with respect to demographic characteristics, clinical manifestations, genetic mutation profiles, and therapeutic management strategies (Tables 4 and 5). One case was not included in the table because the mutation site was not specified (Xie et al., 2022).

**TABLE 4:** Summary of previously reported cases of *IL2RG* mutation combined with *T. marneffei* infection.

Patient	Gender	Age	*T. marneffei* culture	Mutation source	Amino acid changes	Nucleotide changes	Mutation type	Treatment	Prognosis	ACMG classification	References
1	M	8 m	Blood, sputum, BM	Not specified	p.Trp155*	c.464G > A	Nonsense	VCZ, AmB, ITZ, CoSMZ, IVIG	Died	Not specified	Wang et al. (2022)
2	M	4 m	Ascites	Not specified	p.Trp155*	c.464G > A	Nonsense	VCZ, INH, RFP, PZA, LZD, MP, VP-16, CoSMZ, IVIG	Died	Not specified	Wang et al. (2022)
3	M	3 m	Not specified	Maternal	p.Cys62Tyr	c.185G > A	Missense	AmB, Caspofungin	Died	Pathogenic, PS1, PM2, PM4, PP2, PP3, PP4	Zeng et al. (2021), Fan et al. (2025), Fan et al. (2024)
4	M	8 m	Not specified	Maternal	p.Trp155*	c.464G > A	Nonsense	AmB, Itraconazole	Improvement	Pathogenic, PVS1, PS4, PM2, PP2, PP3, PP4	Fan et al. (2025)
5	M	4 m	Not specified	Maternal	p.Trp155*	c.464G > A	Nonsense	Caspofungin, Itraconazole	Died	Pathogenic, PVS1, PS4, PM2, PP2, PP3, PP4	Fan et al. (2025)
6	M	4 m	BM, blood	*de novo*	p.Cys72*	c.216C > A	Nonsense	Imipenem, VRCZ, AmB, linezolid, Levofloxacin, IVIG	Died	Pathogenic, PVS1, PS2, PM2	This study

**TABLE 5:** Clinical characteristics of patients with *IL2RG* mutation and *T. marneffei* infection.

Clinical characteristics	Cases (n/n, %)
Signs and symptoms	
Fever	6/6, 100%
Respiratory tract infection	5/6, 83.33%
Diarrhea	4/6, 66.66%
Skin lesion	4/6, 66.66%
Weight loss	2/6, 33.33%
Hepatosplenomegaly	6/6, 100%
Malnutrition	2/6, 33.33%
Ascites	1/6, 16.66%
Lymphadenopathy	1/6, 16.66%
Anemia	2/6, 33.33%
Hematuria	1/6, 16.66%
Peritonitis	1/6, 16.66%
Significant complication	
HLH	3/6, 50.00%
MODS	4/6, 66.66%
Sepsis	3/6, 50.00%
DIC	2/6, 33.33%
ARDS	2/6, 33.33%
Disseminated tuberculosis	1/6, 16.66%

Most cases exhibited persistent fever, recurrent respiratory infections, hepatosplenomegaly and chronic diarrhea (Table 5). Genetic analysis revealed that 83.33% harbored nonsense mutations in the *IL2RG* gene, with 66.67% carrying the c.464G > A (p.Trp155*) variant. Notably, both this study and recent literature suggest a significantly increased incidence of *T. marneffei* co-infection in patients with X-SCID. Therefore, detection of this pathogen should prompt clinicians to consider underlying immunodeficiency, enabling earlier diagnosis and timely intervention.

## 4 Discussion

This study reports a 4-month-old male infant with chronic diarrhea, recurrent fever, and invasive pulmonary infection. Laboratory investigations revealed profound T-cell deficiency, markedly reduced IgA, IgG, and IgE levels, and co-infections with *M. pneumoniae* and disseminated *T. marneffei*. WES identified a novel *IL2RG* nonsense mutation (c.216C > A, p.Cys72*), producing a hemizygous variant that prematurely truncates the γ-common chain (γc) at cysteine 72. Classified as pathogenic according to ACMG guidelines, family testing confirmed its *de novo* origin. To our knowledge, this is the first global report of this variant. Despite antifungal therapy and immunologic support, the patient showed no improvement, and treatment was withdrawn, leading to death.

According to the HGMD (Pro 2024.4), 278 pathogenic *IL2RG* variants have been reported, including missense, frameshift, nonsense, and splice-site mutations, with missence/nonsence mutations being the most common. The interleukin receptor γc chain is a shared signaling subunit of the IL-2, IL-4, IL-7, IL-9, IL-15, and IL-21 receptor complexes, regulating T, B, and NK cells development and function via the JAK-STAT pathway (Leonard et al., 2019; Habib et al., 2002). In this case, the *IL2RG* mutation likely caused complete structural loss of γc, resulting in classic X-SCID with a T^−^B^+^NK^−^ immunophenotype. Functional assays were not performed due to limited sample availability.

A subset of missense mutations (e.g., exon 8 c.982C > T) (Lim et al., 2019) may partially retain γc-JAK3 binding capacity, presenting as atypical X-SCID with delayed onset or milder infections, but still progressing to immune failure. Our review of reported X-SCID cases with *T. marneffei* co-infection shows that most involve nonsense mutations with rapid clinical deterioration. Therefore, detection of *T. marneffei* in pediatric patients, particularly those with severe or recurrent infections, should prompt early investigation for underlying inborn errors of immunity.

*T. marneffei* is a dimorphic pathogenic fungus endemic to Southeast Asia, which can cause disseminated disease in both immunocompetent and immunocompromised individuals, particularly those with CD4^+^ T cell depletion (Wang et al., 2023; Pruksaphon et al., 2022). It spreads hematogenously and via the lymphatic system, leading to multi-organ abscesses involving the liver, spleen, bone marrow, and skin. Disseminated *T. marneffei* infection occurs predominantly in patients with acquired immunodeficiency, such as HIV/AIDS, but its incidence is also rising among non-HIV immunocompromised populations in parallel with increased use of organ transplantation, immunosuppressants, and targeted therapies (Vanittanakom et al., 2006; Chan et al., 2016). Previous reports have documented concomitant lymphocytopenia and T/NK-cell dysfunction in non-HIV patients with *T. marneffei* infection, all of whom had preexisting immune impairment (Hu et al., 2021).

In this pediatric case, genetically confirmed X-SCID established profound T-cell deficiency as the permissive state for disseminated fungal infection. The child’s progressive anemia and hypodense hepatic lesion were consistent with invasive fungal complications, highlighting the essential role of T-cell-mediated immunity in controlling deep-seated mycoses. Thus, lymphocytopenia in this patient was mechanistically rooted in the *IL2RG* mutation, with *T. marneffei* infection representing a consequence rather than the cause of immune dysregulation.

Flow cytometric immunophenotyping revealed a classic T^−^B^+^NK^−^ immunophenotype, characteristic of X-SCID. Although B lymphocyte counts were elevated, dysfunction of the interleukin-2 receptor gamma chain (γc) disrupted intracellular signaling during T cell development, resulting in T cell developmental arrest and subsequent B-cell functional impairment (Cao et al., 2024). Functional assays could not be performed due to limited blood samples, as the family opted for palliative care following secondary multi-organ failure and disseminated intravascular coagulation.

Mutations in *IL2RG* are the most common genetic cause of X-SCID. Although current reports suggest that the incidence of X-SCID in China is lower than in Western countries; this cannot yet be considered accurate due to the incomplete implementation of newborn screening and limited clinician awareness of IEI manifestations (Puck, 2019; Chen et al., 2024; Kwan et al., 2014). To improve early diagnosis, it is recommended to combine TREC and KREC assays with NGS in newborns from high-risk families. Early genetic analysis diagnosis and timely interventions are crucial, as HSCT performed within 3.5 months of birth yields an 8-year Kaplan-Meier survival rate of 96%, compared with 70% for later transplantation (Railey et al., 2009). Reducing X-SCID mortality in China will require nationwide newborn screening coverage, the establishment of regional IEI referral networks, and individualized treatment pathways guided by ACMG variant interpretation criteria.

## 5 Conclusion

In summary, we report a novel *IL2RG* mutation (NM_000206.3, exon 2, c.216C > A, p.Cys72*) in a patient with a classic X-SCID phenotype. This finding expands the known pathogenic spectrum of *IL2RG* and may improve clinical recognition of related disorders. The patient’s presentation, laboratory data, and co-infection with *T. marneffei* highlight the diagnostic value of integrating phenotypic and molecular analyses in suspected IEI. Review of reported cases indicates that *T. marneffei* infection may serve as an early warning sign of underlying immunodeficiency, warranting prompt evaluation for IEI in affected patients.

## Data Availability

The datasets analyzed during the current study are available from the corresponding author upon reasonable request.

## Abbreviations

IEI, Inborn Errors of Immunity; PIDs, Primary Immunodeficiency Diseases; WHO, World Health Organization; IUIS, International Union of Immunological Societies; NGS, next-generation sequencing; WES, whole-exome sequencing; WGS, whole-genome sequencing; SCID, Severe Combined Immunodeficiency; X-SCID, X-linked severe combined immunodeficiency; *IL2RG*, interleukin-2 receptor gamma chain; HSCT, hematopoietic stem cell transplantation; TRECs, T-cell receptor excision circles; KRECs, kappa-deleting recombination excision circles; ACMG, American College of Medical Genetics and Genomics; PBMCs, Peripheral blood mononuclear cells; HGMD, Human Gene Mutation Database; BM, Bone Marrow; HLH, Hemophagocytic Syndromes; MODS, Multiple Organ Dysfunction Syndrome; VCZ, Voriconazole; AmB, Bmphotericin b; ITZ, Itraconazole; CoSMZ, Compound Sulfamethoxazole Tablets; IVIG, Intravenous Immunoglobulin; INH, Isoniazid; RFP, Rifampicin; PZA, Pyrazinamide; LZD, Linezolid; MP, Methylprednisolone; VP-16, Etoposide; ARDS, Respiratory Distress Syndrome; DIC, Disseminated Intravascular Coagulation; VRCZ, Voriconazole; WBC, white blood cell.
